# Goreisan-mediated suppression of chemokine and cytokine production in astrocytes under AQP4-expressing conditions

**DOI:** 10.3389/fnins.2026.1860290

**Published:** 2026-08-31

**Authors:** Kazuhito Murakami, Tomofumi Shimizu, Toshitaka Kido, Yoichiro Isohama

**Affiliations:** 1 Laboratory of Applied Pharmacology, Faculty of Pharmaceutical Sciences, Tokyo University of Science, Tokyo, Japan; 2 TSUMURA Kampo Research Laboratories, Research & Development Division, TSUMURA & CO., Ibaraki, Japan

**Keywords:** aquaporin-4, astrocytes, ERK signaling, Goreisan, neuroinflammation

## Abstract

**Aim:**

Aquaporin-4 (AQP4), predominantly expressed in astrocytes, has been implicated in neuroinflammation through the amplification of cytokine and chemokine production. Goreisan (GRS), a traditional Japanese herbal medicine, modulates AQP4-related water homeostasis. However, its effects on AQP4-associated inflammatory processes remain unclear. This study aimed to elucidate the anti-inflammatory effects of GRS, with particular emphasis on inflammatory responses observed under AQP4-expressing conditions.

**Methods:**

*In vitro* experiments were conducted using primary cultured astrocytes and AQP4-overexpressing NIH-3T3 fibroblasts stimulated with lipopolysaccharide (LPS) or tumor necrosis factor-alpha (TNF-α), with or without GRS. Cytokine and chemokine expression levels were assessed by quantitative reverse transcription polymerase chain reaction, whereas ERK phosphorylation was evaluated by Western blotting. An *in vivo* mouse model of LPS-induced neuroinflammation was employed to assess the effects of orally administered GRS on inflammatory markers and glial activation. The contributions of individual herbal components were also examined.

**Results:**

GRS suppressed LPS- and TNF-α-induced cytokine and chemokine mRNA expression in primary astrocytes and AQP4-expressing NIH-3T3 cells. *In vivo*, GRS administration reduced inflammatory cytokine expression and attenuated glial activation in the brain. GRS attenuated ERK phosphorylation under AQP4-expressing conditions, in parallel with reduced chemokine mRNA expression. Furthermore, *Cinnamomi cortex*, a component of GRS, was identified as a major contributor to these anti-inflammatory effects.

**Conclusion:**

These findings indicate that the effects of GRS on inflammatory gene expression and ERK activation are associated with AQP4-expressing conditions. However, the present data do not establish AQP4 as a direct or primary molecular target of GRS. Therefore, GRS may provide a potential pharmacological approach for modulating inflammatory responses observed under AQP4-expressing conditions, although further studies are required to confirm its therapeutic relevance.

## 1 Introduction

Aquaporin-4 (AQP4) is the predominant water channel in the central nervous system (CNS) and is highly enriched in astrocytic endfeet facing cerebral microvessels and the glia limitans. Through this polarized localization, AQP4 plays an essential role in brain water homeostasis (Fukuda and Badaut, 2012), edema formation and resolution, and fluid exchange within the neurovascular unit. In addition to its canonical function as a water channel, increasing evidence suggests that AQP4 is also associated with astrocytic responses under pathological conditions, including neuroinflammation (Benarroch, 2007; Lan et al., 2016; Xiao and Hu, 2014). Altered AQP4 expression or localization has been reported in various CNS disorders, such as neuromyelitis optica spectrum disorder, multiple sclerosis, traumatic or ischemic brain injury, and chronic neurodegenerative diseases (Mader and Brimberg, 2019; Nataf, 2020; Sinclair et al., 2007; Vandebroek and Yasui, 2020).

Astrocytes are central regulators of neuroinflammatory responses. Upon inflammatory stimulation, astrocytes express cytokines and chemokines that influence surrounding glial cells, neurons, and vascular components. Astrocyte-derived inflammatory mediators can promote microglial activation and contribute to the maintenance of neuroinflammatory signaling. Previous studies have suggested that AQP4 expression is associated with enhanced inflammatory responses in astrocytes, whereas AQP4 deficiency or pharmacological blockade can attenuate inflammatory signaling in several experimental models (Dai et al., 2018; Nicosia et al., 2019; Salman et al., 2022). These findings suggest that AQP4 may contribute not only to water transport but also to inflammatory amplification in astrocytes. However, pharmacological approaches capable of modulating AQP4-associated inflammatory responses remain limited.

Goreisan (GRS) is a traditional Japanese Kampo medicine that has long been used for symptoms and disorders associated with fluid imbalance, including edema, dizziness, and headache. Experimental studies, including our previous work, have shown that GRS can modulate AQP4-related water transport and attenuate brain edema *in vivo* (Shimizu et al., 2023). These findings raise the possibility that GRS may also influence inflammatory responses associated with AQP4-expressing conditions. However, whether GRS affects astrocytic inflammatory signaling and how this effect relates to AQP4 expression have not been fully clarified.

Hence, this study aimed to investigate the effects of GRS on inflammatory gene expression using *in vitro* and *in vivo* models, with particular emphasis on responses observed under AQP4-expressing conditions. We also examined ERK phosphorylation and the contribution of individual herbal components, including Cinnamomi cortex.

## 2 Material and methods

### 2.1 Reagents

A spray-dried GRS extract (lot no. 2180017010) was provided by TSUMURA & CO. (Tokyo, Japan) and used in all experiments. GRS consisted of *Alismatis Rhizoma* (4 g; rhizome of *Alisma orientale* Juzep), *Atractylodis Lanceae Rhizoma* (3 g; rhizome of *Atractylodes lancea* DC.), *Polyporus* (3 g; sclerotium of *Polyporus umbellatus* Fries), Hoelen (3 g; sclerotium of *Poria cocos* Wolf), and Cinnamomi Cortex (1.5 g; cortex of *Cinnamomum cassia* Blume). A rabbit polyclonal anti-AQP4 antibody was obtained from Sigma-Aldrich (St. Louis, MO, USA). Rabbit anti-phospho-p44/42 MAPK (ERK1/2; Thr202/Tyr204) and anti-p44/42 MAPK (ERK1/2) antibodies were purchased from Cell Signaling Technology (Danvers, MA, USA). U0126, cinnamaldehyde, and trans-cinnamic acid were purchased from FUJIFILM Wako Pure Chemical Corporation (Osaka, Japan).TGN-020 was purchased from MedChemExpress (NJ, USA). Lipopolysaccharide (LPS) from *Escherichia coli* O111:B4, purified by phenol extraction, was purchased from Sigma-Aldrich (St. Louis, MO, USA; Cat. No. L2630).

### 2.2 Cell culture

Primary astrocyte-enriched cultures were prepared from the cerebral cortices of postnatal day 2–3 rats as described previously with minor modifications (McCarthy and de Vellis, 1980). Briefly, cerebral cortices were dissected, and the meninges were carefully removed. The tissues were enzymatically dissociated with trypsin and mechanically triturated to obtain a single-cell suspension. Cells were plated on poly-D-lysine-coated culture dishes and maintained in Dulbecco’s modified Eagle’s medium (DMEM) supplemented with 10% fetal bovine serum (FBS) and penicillin/streptomycin at 37°C in a humidified atmosphere containing 5% CO_2_. The culture medium was changed every 2–3 days. After the cultures reached confluence, non-astrocytic cells were removed by shaking, and the remaining astrocyte-enriched cultures were used for experiments. NIH-3T3 fibroblasts were maintained in DMEM supplemented with 10% FBS and penicillin-streptomycin. All cells were incubated at 37°C in a humidified atmosphere containing 5% CO_2_. NIH-3T3 cells were used as a heterologous AQP4-expression system, not as an astrocyte model, because these cells exhibit little endogenous AQP4 expression and allow comparison between empty vector-transfected and AQP4-expressing cells under the same cellular background.

### 2.3 Transient expression of AQP4 in NIH-3T3 cells

NIH-3T3 cells were cultured in 12-well plates and transiently transfected with an AQP4 expression plasmid or the corresponding empty vector using TransIT-LT1 Transfection Reagent (Mirus Bio LLC, Madison, WI, USA) according to the manufacturer’s instructions. The plasmid DNA was diluted in 100 μL of Opti-MEM I Reduced Serum Medium (Thermo Fisher Scientific, Waltham, MA, USA). TransIT-LT1 Transfection Reagent was then added at a ratio of 3 μL of reagent per 1 μg of plasmid DNA, and the mixture was incubated for 15–30 min at room temperature to allow formation of DNA–reagent complexes. The transfection mixture was subsequently added dropwise to each well.

For the plasmid dose-response experiment, cells were transfected with 1, 2, or 4 μg of the AQP4 expression plasmid per well. Accordingly, 3, 6, or 12 μL of TransIT-LT1 Transfection Reagent, respectively, was used per well. In the subsequent experiments, cells were transfected with 4 μg per well of either the AQP4 expression plasmid or the corresponding empty vector using 12 μL of TransIT-LT1 Transfection Reagent. At 24 h after transfection, the cells were stimulated with LPS or TNF-α. Expression of AQP4 in the transfected cells was confirmed by Western blotting.

### 2.4 GRS treatment

GRS was dissolved in dimethyl sulfoxide, sonicated for 20 min, and subsequently centrifuged (3,000×g, 25°C, 5 min). The resulting supernatant was used for all experiments. Cells were treated with GRS (0.01, 0.03, or 0.1 mg/mL) in the presence of LPS (100 ng/mL for astrocytes or 10 μg/mL for NIH-3T3 cells) or TNF-α (40 ng/mL) for an additional 1 or 6 h. For experiments using individual crude drug components, each component was applied at a concentration equivalent to its proportion in 0.1 mg/mL GRS. The GRS concentrations used in the *in vitro* experiments were selected based on our previous studies demonstrating pharmacological effects of GRS in cell-based assays (Murakami et al., 2021a,b). For experiments examining the chemical constituents of Cinnamomi cortex, cells were treated with cinnamaldehyde or trans-cinnamic acid at concentrations ranging from 5 to 50 μM (Kim et al., 2020).

### 2.5 Real-time quantitative reverse transcription-polymerase chain reaction

The cells were lysed in 1 ml of RNAiso Plus^®^ reagent (Takara Bio Inc., Shiga, Japan) and homogenized on ice, after which total RNA was extracted following the manufacturer’s instructions. Reverse transcription was performed using PrimeScript^®^ RT Master Mix (Takara Bio Inc.). Real-time polymerase chain reaction (PCR) was conducted using TB-Green^®^ Premix Ex Taq™ (Takara Bio Inc.) on a Chromo 4™ real-time PCR analysis system (Bio-Rad Laboratories, Tokyo, Japan). All samples were analyzed in duplicate. The thermal cycling conditions comprised an initial polymerase activation step at 95°C for 3 min, followed by 40 cycles of denaturation at 95°C for 15 s and combined annealing and extension at 60 °C for 1 min. Quantification was performed by determining the threshold cycle [Ct], defined as the amplification cycle at which the target PCR product was first detected. Relative gene expression was calculated using the comparative Ct method.

### 2.6 Western blotting

Cells were washed with phosphate-buffered saline (PBS, pH 7.4) and subsequently lysed in buffer [50 mM Tris–HCl, pH 7.5, 150 mM NaCl, 5 mM ethylenediaminetetraacetic acid, 1% (v/v) NP-40, 0.1% (w/v) sodium dodecyl sulfate (SDS), 0.5% (w/v) deoxycholate, and 1% (v/v) protease inhibitor cocktail] for 30 min at 4°C. Protein concentrations were determined using the bicinchoninic acid assay, with bovine serum albumin as the standard. Ten micrograms of protein were separated by 10% SDS–polyacrylamide gel electrophoresis and transferred onto a polyvinylidene fluoride membrane. The membrane was blocked with 5% skim milk in Tris-buffered saline containing 0.1% Tween-20 (TBS-T) for 1 h at room temperature. Subsequently, the membrane was incubated with affinity-purified rabbit anti-phospho-p44/p42 MAPK (1:1,000), anti-p44/p42 MAPK (1:1,000), or anti-AQP4 (1:1,000) antibodies at 4°C for 8 h. The membrane was washed with TBS-T and incubated with horseradish peroxidase-conjugated donkey anti-rabbit IgG for 1 h at room temperature. Immunoreactive bands were visualized using enhanced chemiluminescent reagents (Thermo Fisher Scientific, Waltham, MA, USA).

### 2.7 *In vivo* model

Male C57BL/6J mice (8–10 weeks old) were used in this study. Mice were housed in plastic cages in a room with controlled air temperature and pressure under a 12-h light/dark cycle (lights on between 08:00 and 20:00), with free access to food and water.Mice were anesthetized by intraperitoneal administration of a mixture of medetomidine, midazolam, and butorphanol (MMB) (Kawai et al., 2011; Tashiro and Tohei, 2022). The anesthetic mixture consisted of medetomidine (0.3 mg/kg), midazolam (4 mg/kg), and butorphanol (5 mg/kg), and was administered at a volume of 0.1 mL per 10 g body weight. To prevent anesthesia-induced hypothermia, mice were placed on a heating pad during the procedure and maintained under warming conditions until recovery of the righting reflex. After completion of the procedure, anesthesia was reversed by intraperitoneal injection of atipamezole at a dose of 0.75 mg/kg.

At the end of the experiment, mice were euthanized by cervical dislocation. All efforts were made to minimize animal suffering and to reduce the number of animals used.

All animal procedures were approved by the Tokyo University of Science Animal Care and Use Committee (approval reference No. Y20010, Y22004) and were performed in accordance with institutional guidelines.

Intracerebroventricular (i.c.v.) injection was performed under anesthesia induced by intraperitoneal administration of MMB, as described above. Mice were placed in a stereotaxic frame, and a small burr hole was made in the skull. A microsyringe was used to inject the solution (LPS, 12.5 μg) into the lateral ventricle at the following coordinates relative to bregma: [AP -0.5 mm, ML +1.0 mm, DV -3.2 mm]. The injection volume was 5 μL per mouse and was delivered at a constant rate. After injection, the needle was left in place for several minutes to prevent reflux and then slowly withdrawn.

After completion of the procedure, anesthesia was reversed by intraperitoneal injection of atipamezole (0.75 mg/kg). Mice were maintained on a heating pad until recovery of the righting reflex.

GRS was orally administered at a dose of 3 g/kg as a single dose 30 min before LPS injection. This dose was selected based on our previous *in vivo* study that demonstrated pharmacological effects of oral GRS on brain edema and AQP4-related responses (Shimizu et al., 2023).

At 6 h after LPS injection, mice were euthanized, and brain tissues were collected for qPCR, immunohistochemical, and western blot analyses.

### 2.8 Histological analysis and immunohistochemistry

Brain tissues were fixed with 4% paraformaldehyde, cryoprotected, embedded, and sectioned using a cryostat. Coronal brain sections were prepared at a thickness of 20 μm. Sections were washed with PBS and permeabilized with 0.1% Triton X-100 in PBS. After blocking with 3% BSA for 30 min at room temperature, sections were incubated with anti-Iba-1 antibody (1:500) and anti-GFAP antibody (1:500) overnight at 4°C. After washing with PBS, sections were incubated with fluorophore-conjugated secondary antibodies (1:500) for 60 min at room temperature. The sections were mounted, and fluorescence images were acquired using a BZ-9000 fluorescence microscope (Keyence, Osaka, Japan).

### 2.9 Statistical analysis

Statistical analyses were performed using GraphPad Prism. Data are presented as the mean ± SEM. Comparisons among multiple groups with one independent variable were performed using one-way ANOVA followed by Turkey’s multiple comparisons test. Experiments involving two independent variables, such as AQP4 expression status and drug treatment, were analyzed using two-way ANOVA followed by Turkey’s or Sidak’s multiple comparisons test, as appropriate. For time-course experiments involving repeated measurements, repeated-measures ANOVA was used. A value of *p* < 0.05 was considered statistically significant.

## 3 Results

### 3.1 GRS suppression of chemokine and cytokine expression in primary astrocytes

**FIGURE 1:**
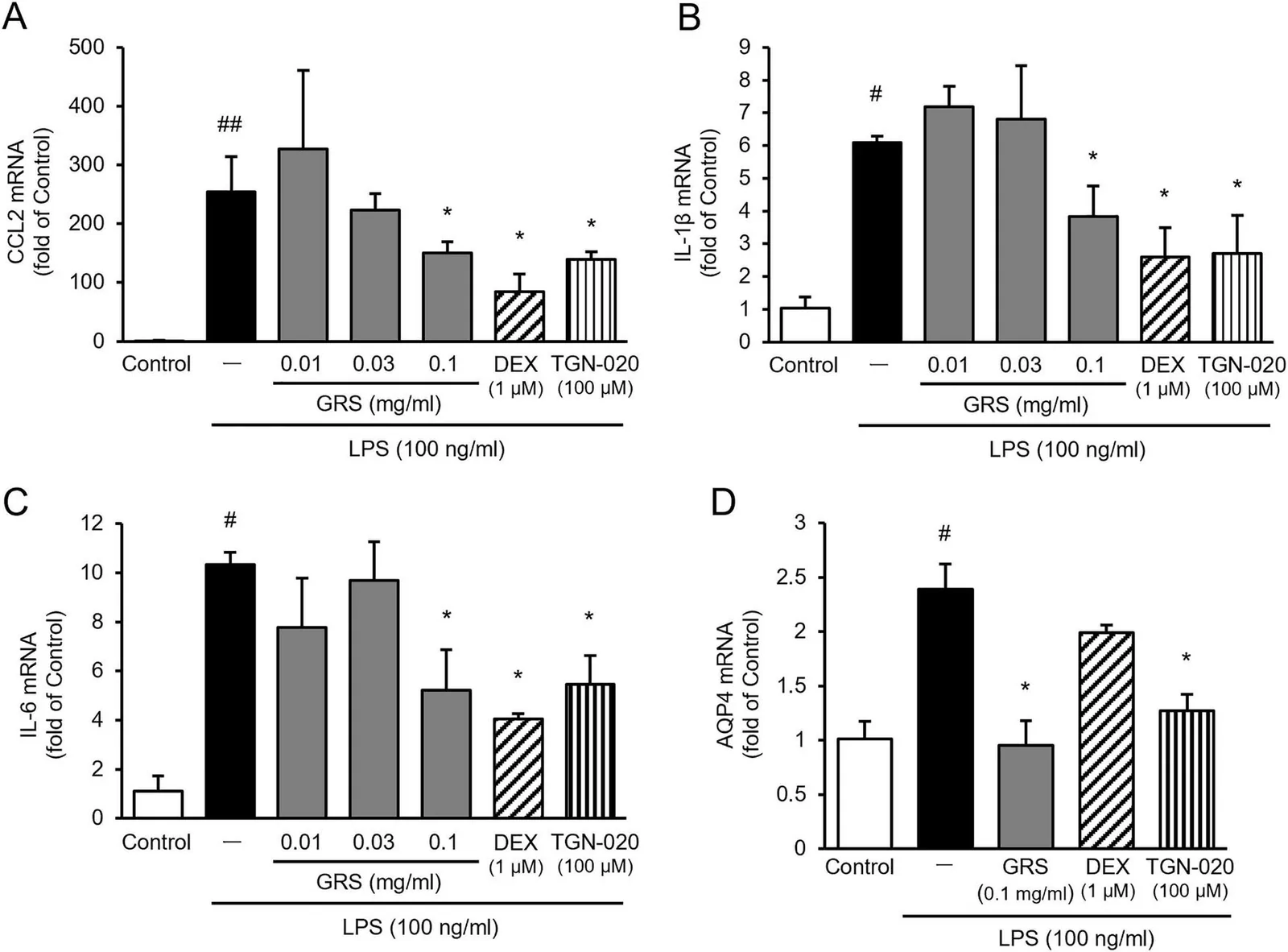
GRS suppressed chemokine and cytokine expression in primary cultured astrocytes. Primary cultured astrocytes were stimulated with LPS (100 ng/ml) in the presence or absence of Goreisan (GRS; 0.01–0.1 mg/ml), dexamethasone (DEX; 1 μM), and TGN-020 (100 μM). The mRNA expression levels of CCL2 **(A)**, IL-1β **(B)**, IL-6 **(C)**, and AQP4 **(D)** were measured by RT-qPCR. Data are presented as the mean ± SEM (*n* = 3). Statistical analysis was performed using one-way analysis of variance (ANOVA), followed by Turkey’s multiple comparisons test. ^##^*P* < 0.01 and ^#^*P* < 0.05 vs. control; *P < 0.05 vs. the LPS-treated group. LPS, lipopolysaccharide; AQP4, aquaporin-4; IL, interleukin; RT-qPCR, quantitative reverse transcription-polymerase chain reaction.

To evaluate the anti-inflammatory effects of GRS, we first examined its effects on cytokine and chemokine expression in LPS-stimulated primary cultured astrocytes. As shown in Figures 1A–C, LPS-treatment significantly upregulated CCL2, IL-1β and IL-6 mRNA levels. GRS treatment suppressed this increase in a dose-dependent manner (0.01–0.1 mg/ml). These suppressive effects on cytokine and chemokine expression were similar to those of dexamethasone (DEX). Interestingly, TGN-020, a specific AQP4 inhibitor, suppressed the expression of the cytokines. We also analyzed AQP4 expression in cultured astrocytes. LPS stimulation increased AQP4 mRNA levels (Figure 1D), and this increase was significantly suppressed by 0.1 mg/ml GRS and by TGN-020, but not by DEX (Figure 1D). These data suggest that the effects of GRS and TGN-020 on LPS-induced gene expression may occur through a mechanism different from that of general anti-inflammatory agents.

### 3.2 AQP4 overexpression enhancing chemokine expression, suppressed by GRS

**FIGURE 2:**
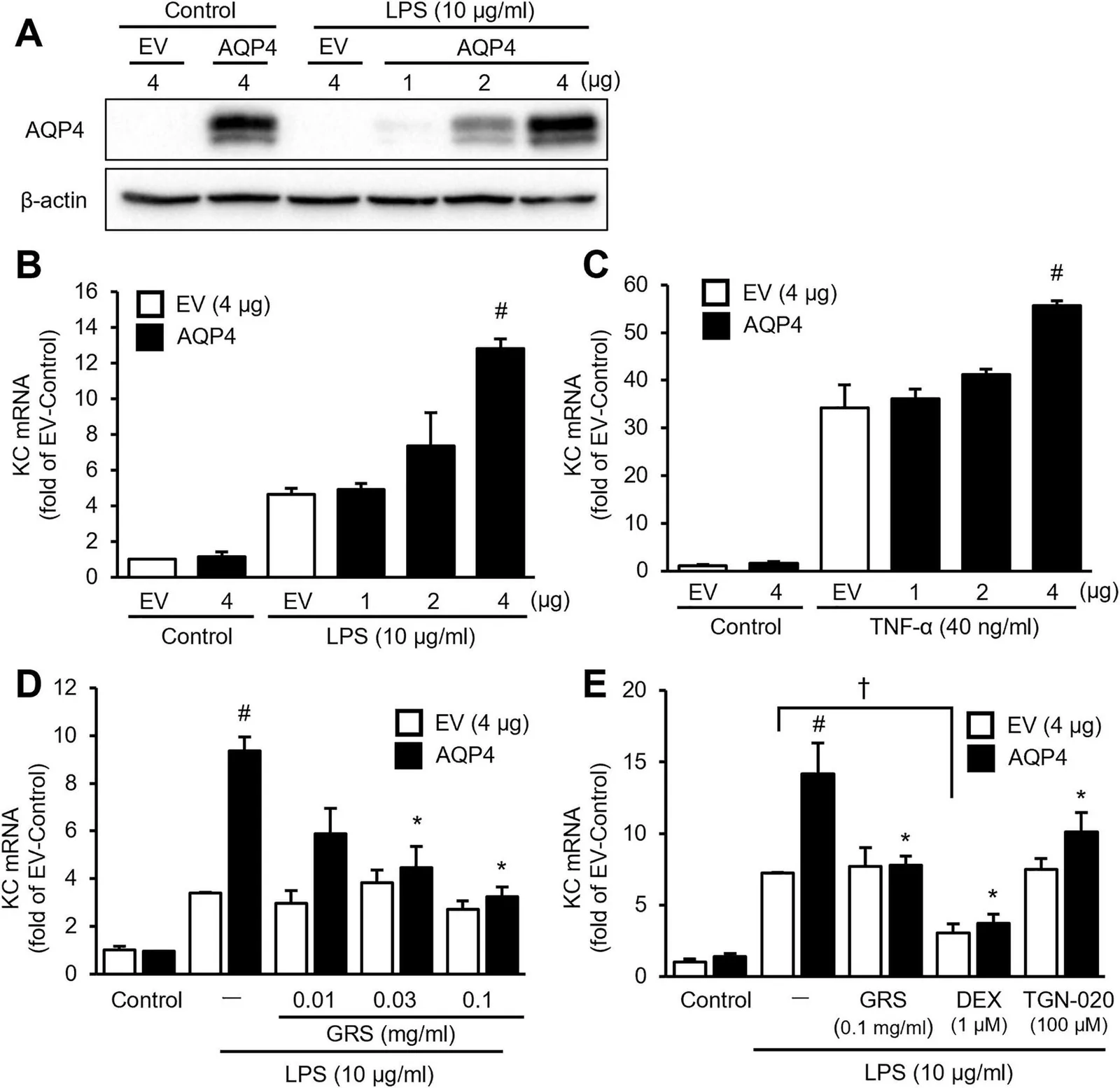
AQP4 expression was associated with increased chemokine mRNA expression, which was attenuated by GRS. **(A–C)** NIH-3T3 cells were transfected with increasing amounts of AQP4 plasmid (1–4 μg). Cells were stimulated with LPS (10 μg/ml) or TNF-α (40 ng/ml). KC mRNA expression was measured using RT-qPCR. **(D, E)** NIH-3T3 cells expressing AQP4 (4 μg) were stimulated with LPS (10 μg/ml) in the presence or absence of Goreisan (GRS; 0.01–0.1 mg/ml), dexamethasone (DEX; 1 μM) and TGN-020 (100 μM). KC mRNA expression was measured using RT-qPCR. Data are presented as mean ± SEM (*n* = 3). Statistical analysis was performed using two-way ANOVA followed by Turkey’s multiple comparison test. ^#^*P* < 0.05 vs. EV-control; *P < 0.05 vs. LPS- treated group; ^†^*P* < 0.05. GRS, Goreisan; EV, empty vector; LPS, lipopolysaccharide; AQP4, Aquaporin-4; TNF, tumor necrosis factor; RT-qPCR, quantitative reverse transcription-polymerase chain reaction; KC, keratinocyte chemoattractant.

To examine whether AQP4 expression was associated with the inflammatory response, we transfected NIH-3T3 fibroblasts (i.e., cells that did not naturally express AQP4) with AQP4 or an empty vector (EV). Upon stimulation with TNF-α (40 ng/ml) or LPS (10 μg/ml), AQP4-overexpressing cells showed enhanced keratinocyte chemoattractant (KC) mRNA expression compared to EV-transfected controls (Figures 2A–C). GRS treatment dose-dependently reduced LPS-induced KC expression in AQP4-expressing cells (Figure 2D), but did not affect EV-transfected cells. This effect was comparable to that of TGN-020 and DEX (Figure 2E), indicating that GRS attenuated the enhanced response observed under AQP4-expressing conditions.

### 3.3 GRS suppression of chemokine mRNA expression under AQP4-expressing conditions, accompanied by attenuation of ERK phosphorylation

**FIGURE 3:**
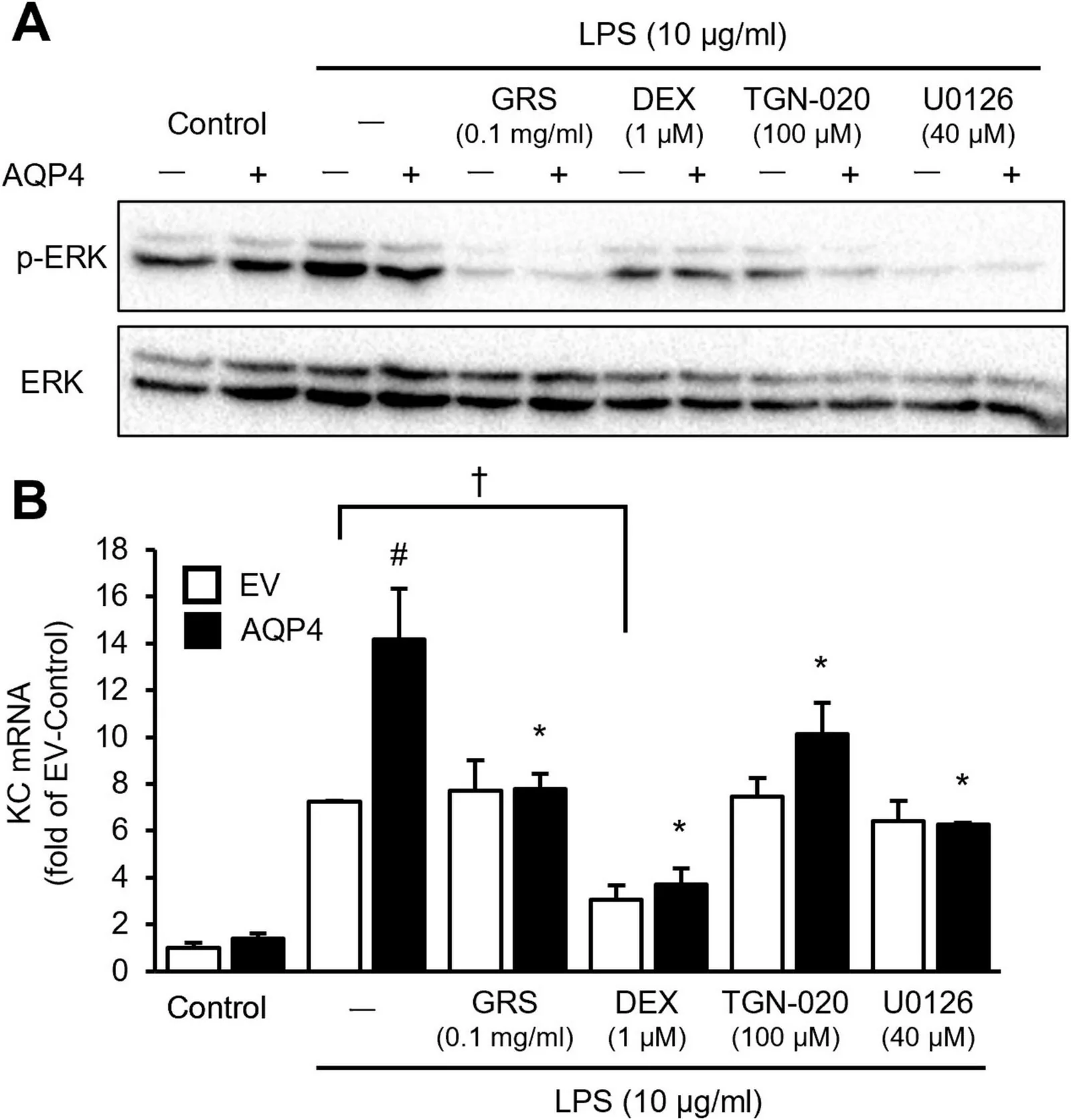
GRS suppressed chemokine mRNA expression and ERK phosphorylation under AQP4-expressing conditions. NIH-3T3 cells expressing AQP4 were stimulated with LPS (10 μg/ml) in the presence or absence of Goreisan (GRS; 0.01–0.1 mg/ml), dexamethasone (DEX; 1 μM), TGN-020 (100 μM) and U0126 (40 μM). ERK phosphorylation was measured using western blot analysis **(A)**. KC mRNA expression was measured using RT-qPCR **(B)**. Data are presented as mean ± SEM (*n* = 3). Statistical analysis was performed using two-way ANOVA followed by Turkey’s multiple comparison test. ^#^*P* < 0.05 vs. EV-control; *P < 0.05 vs. LPS-treated group; ^†^*P* < 0.05. GRS, Goreisan; EV, empty vector; LPS, lipopolysaccharide; KC, keratinocyte chemoattractant; RT-qPCR, quantitative reverse transcription-polymerase chain reaction; AQP4, Aquaporin-4; TNF, tumor necrosis factor.

To explore the underlying mechanism, we examined the ERK signaling pathway. As shown in Figure 3A, LPS stimulation induced ERK phosphorylation in the AQP4-overexpressing NIH-3T3 cells. GRS, TGN-020, and U0126, a MEK inhibitor, suppressed this phosphorylation. In addition, these treatments reduced KC mRNA expression (Figure 3B), suggesting that ERK activation was associated with chemokine mRNA expression under AQP4-expressing conditions.

### 3.4 Cinnamomi cortex and components contributing to the anti-inflammatory effect of GRS

**FIGURE 4:**
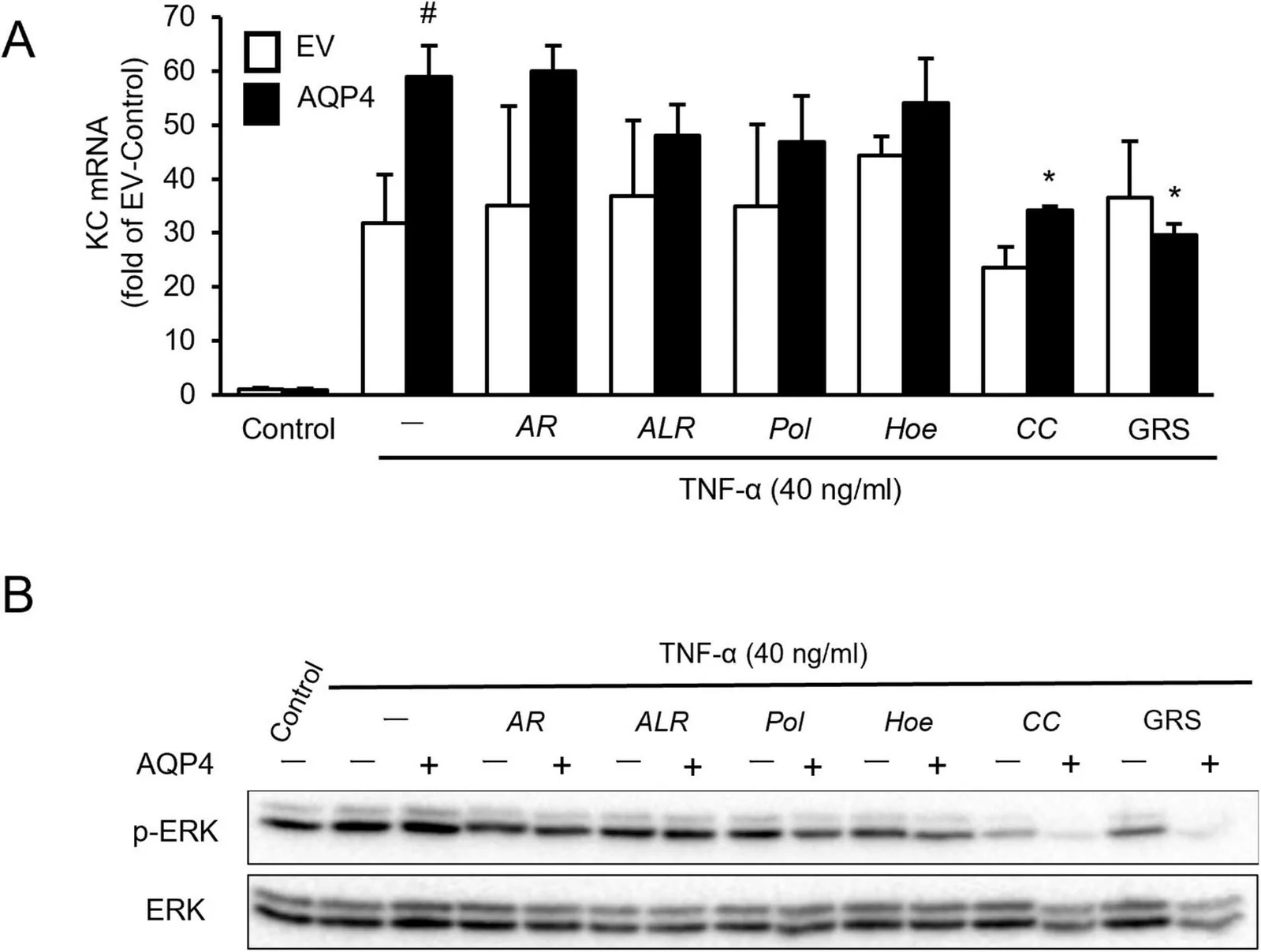
Cinnamomi cortex reduced chemokine mRNA expression under AQP4-expressing conditions. NIH-3T3 cells expressing AQP4 were stimulated with TNF-α (40 ng/ml) in the presence or absence of Goreisan (GRS; 0.1 mg/ml) or its individual crude drug components. Each component was applied at concentrations equivalent to its proportion in GRS at 0.1 mg/mL. KC mRNA expression was measured using RT-qPCR **(A)**. ERK phosphorylation was measured by western blotting **(B)**. AR: Alismatis Rhizoma (rhizome of *Alisma orientale*), ALR: Atractylodis Lanceae Rhizoma (rhizome of *Atractylodes lancea*), Pol: Polyporus (sclerotium of *Polyporus umbellatus*), Hoe: Hoelen (sclerotium of *Poria cocos*), CC: Cinnamomi cortex (bark of *Cinnamomum cassia*). Data are presented as mean ± SEM (*n* = 3). Statistical analysis was performed using two-way ANOVA followed by Turkey’s multiple comparison test. ^#^*P* < 0.05 vs. EV-control; *P < 0.05 vs. TNF-α-treated group. GRS, Goreisan; EV, empty vector; KC, keratinocyte chemoattractant; RT-qPCR, quantitative reverse transcription-polymerase chain reaction; AQP4, Aquaporin-4; TNF, tumor necrosis factor.

We examined the individual components of GRS to identify its active ingredients. In AQP4-overexpressing cells stimulated with TNF-α, only Cinnamomi cortex significantly reduced KC mRNA levels (Figure 4A) and suppressed ERK phosphorylation (Figure 4B), suggesting Cinnamomi cortex is a major contributor to the anti-inflammatory effect of GRS.

**FIGURE 5:**
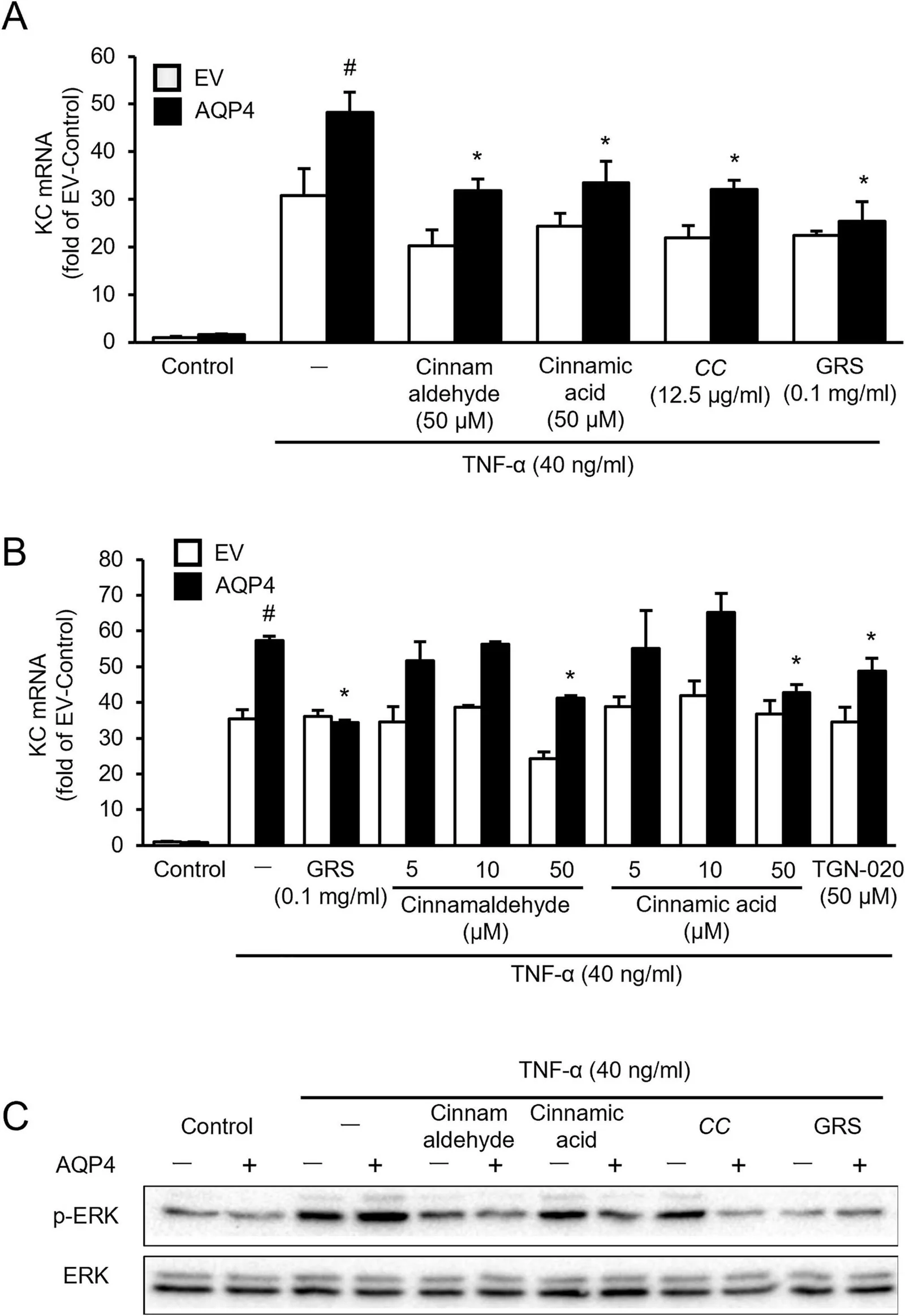
Cinnamaldehyde and cinnamic acid reduced chemokine mRNA expression under AQP4-expressing conditions. NIH-3T3 cells expressing AQP4 were stimulated with TNF-α (40 ng/ml) in the presence or absence of cinnamaldehyde (5–50 μM), cinnamic acid (5–50 μM) and Cinnamomi cortex (12.5 μg/ml). KC mRNA expression was measured using RT-qPCR **(A, B)**. ERK phosphorylation was measured using western blot analysis **(C)**. CC: Cinnamomi cortex (bark of *Cinnamomum cassia*). Data are presented as mean ± SEM (*n* = 3). Statistical analysis was performed using two-way ANOVA followed by Turkey’s multiple comparison test. ^#^*P* < 0.05 vs. EV-control; *P < 0.05 vs. TNF-α-treated group. EV, empty vector; KC, keratinocyte chemoattractant; RT-qPCR, quantitative reverse transcription-polymerase chain reaction; AQP4, Aquaporin-4; TNF, tumor necrosis factor.

We then examined the effects of cinnamaldehyde and cinnamic acid, the major components of Cinnamomi cortex. In addition to GRS and Cinnamomi cortex, both cinnamaldehyde and cinnamic acid suppressed KC mRNA expression without altering its expression in EV-transfected control cells (Figures 5A, B). These compounds also inhibited LPS-induced ERK phosphorylation in AQP4-expressing cells (Figure 5C). These data indicated that the anti-inflammatory effects of GRS were, at least in part, due to cinnamaldehyde and cinnamic acid.

### 3.5 GRS suppression of lPS-induced brain inflammation *in vivo*

**FIGURE 6:**
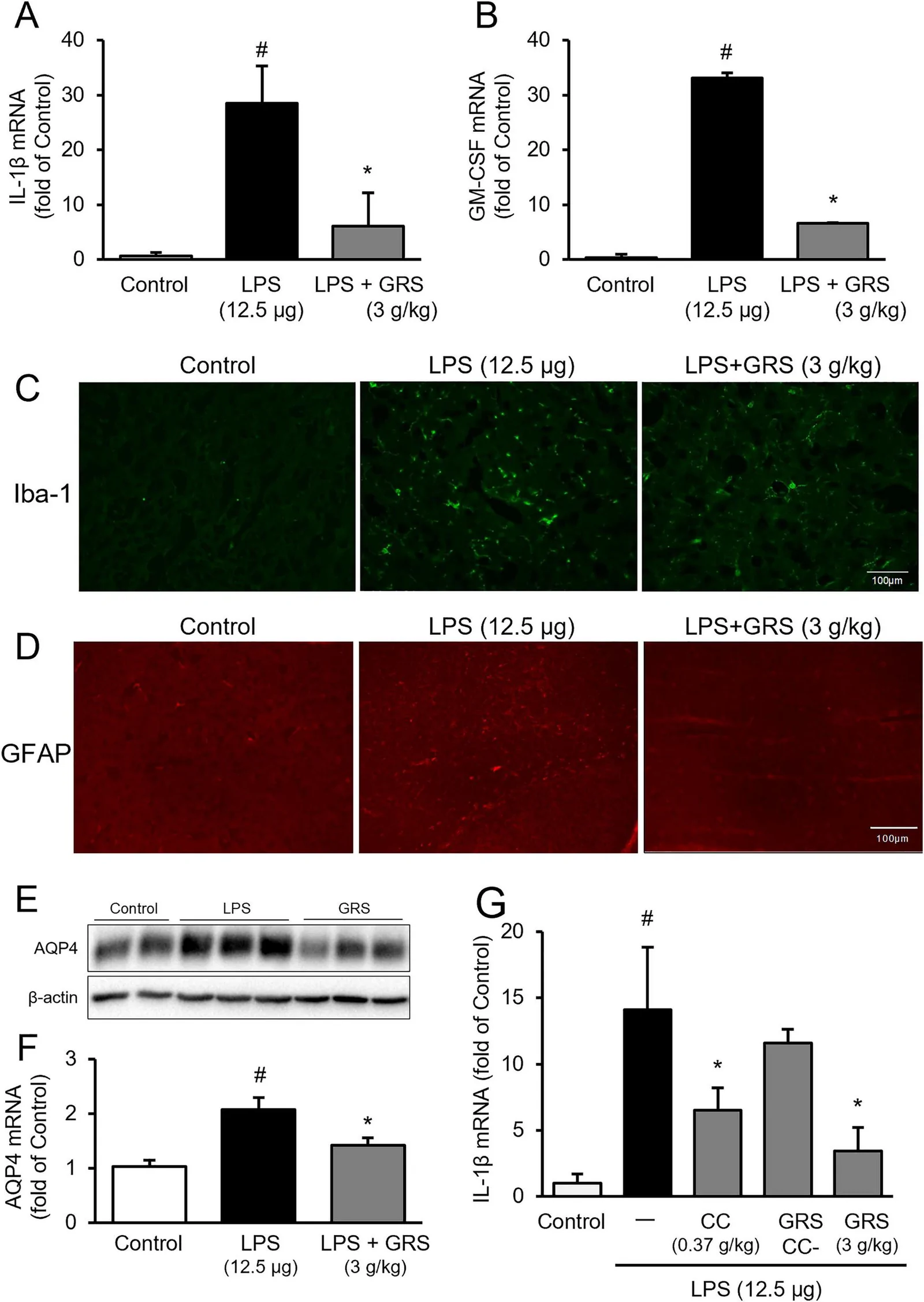
Goreisan attenuated LPS-induced inflammatory responses in the brain *in vivo*. C57BL/6J mice were injected with LPS (12.5 μg/mouse, i.c.v.) and orally administered GRS (3 g/kg, p.o.). The mRNA expression levels of IL-1β **(A)** and GM-CSF **(B)** were measured using RT-qPCR. Protein expression of Iba-1 **(C)** and GFAP **(D)** was evaluated by immunofluorescence staining. These images are presented as representative staining patterns to support the assessment of microglial and astrocytic activation. AQP4 protein expression was measured by western blot analysis **(E)**, and AQP4 mRNA expression was measured by RT-qPCR **(F)**. C57BL/6J mice were injected with LPS (12.5 μg/mouse, i.c.v.) and administered Cinnamomi cortex (0.37 g/kg, p.o.), Cinnamomi cortex-deleted formulation (GRS CC-; 2.63 g/kg), or GRS (3 g/kg, p.o.). IL-1β mRNA expression was measured by RT-qPCR **(G)**. Data are presented as the mean ± SEM (*n* = 4). Statistical analysis was performed using one-way analysis of variance (ANOVA), followed by Turkey’s multiple comparisons test. ^#^*P* < 0.05 vs. control; *P < 0.05 vs. the LPS-treated group. CC, Cinnamomi cortex (bark of Cinnamomum cassia); GRS CC-, GRS CC- consisted of the four remaining crude drug extracts at doses equivalent to those contained in GRS at 3 g/kg, with CC omitted. GRS, Goreisan; LPS, lipopolysaccharide; RT-qPCR, quantitative reverse transcription-polymerase chain reaction; AQP4, aquaporin-4; IL, interleukin; i.c.v., intracerebroventricular; GM-CSF, granulocyte–macrophage colony-stimulating factor; GFAP, glial fibrillary acidic protein; Iba-1, ionized calcium-binding adaptor molecule 1.

Finally, to confirm its *in vivo* relevance, we used a mouse model of LPS-induced neuroinflammation. LPS administration significantly upregulated IL-1β and GM-CSF mRNA levels in the brain, consistent with the effects of neuroinflammation. Oral administration of GRS (3 g/kg) markedly reduced the expression of these inflammatory markers (Figures 6A, B). Representative immunofluorescence images suggested that intracerebroventricular LPS injection increased Iba-1 and GFAP immunoreactivity and that GRS treatment attenuated these LPS-induced changes (Figures 6C, D). GRS also suppressed AQP4 expression (Figures 6E, F). Furthermore, the anti-inflammatory effect of GRS was attenuated when Cinnamomi cortex was excluded from the formulation, emphasizing its essential role (Figure 6G).

## 4 Discussion

In this study, we demonstrated that GRS suppresses LPS-induced inflammatory responses both *in vivo* and *in vitro* (Figures 1, 2, and 6). Recent studies have suggested that AQP4, a water channel protein highly expressed in astrocytes (Fukuda and Badaut, 2012; Ikeshima-Kataoka, 2016; Michinaga and Koyama, 2021), is associated with neuroinflammatory responses beyond its classical role in water transport (Fukuda and Badaut, 2012; Hsu et al., 2015). In the present study, AQP4-expressing cells showed greater inflammatory stimulus-induced KC mRNA expression than EV-transfected cells, and GRS attenuated these responses under the AQP4-expressing conditions. These findings support an association between AQP4 expression status and the magnitude of inflammatory gene expression, while remaining within the limits of the current experimental design.

Neuroinflammation is a highly orchestrated process that involves complex interactions between glial cells, primarily astrocytes and microglia (Garland et al., 2022; Jha et al., 2019). Astrocytes maintain CNS homeostasis, whereas microglia function as immune sentinels responding to tissue injury or infection. Upon inflammatory stimulation, both cell types release pro-inflammatory cytokines and chemokines such as TNF-α, IL-1β, IFN-γ, CCL2, and CXCL10 (Garland et al., 2022; Kirkley et al., 2017; Tanuma et al., 2006). Astrocyte-derived mediators, including CCL2, have been implicated in microglial activation and the persistence of neuroinflammatory signaling (Dubový et al., 2018; He et al., 2016). Conversely, microglia-derived IL-1α, TNF, and C1q promote neurotoxic A1 astrocyte conversion through the C3/C3aR/nuclear factor-kappa B (NF-κB) pathway, contributing to chronic neurodegeneration (Liddelow et al., 2017). Given this bidirectional astrocyte–microglia communication, the present findings may be relevant to inflammatory conditions in which astrocyte-related signaling is enhanced. AQP4 has been shown to participate in astrocytic cytokine secretion, inflammatory amplification, and glial interactions through the activation of intracellular pathways such as NF-κB and MAPKs (Dai et al., 2018; Hsu et al., 2015; Tang et al., 2022). Furthermore, activation of the Toll-like receptor 4 (TLR4)/NF-κB-p65 pathway via aquaporins may enhance astrocytic activation and influence microglial polarization under inflammatory conditions (Dai et al., 2018; Fukuda and Badaut, 2012). Consistent with previous studies, overexpression of AQP4 was associated with a greater response to LPS and TNF-α in NIH-3T3 cells (Figure 2).

The anti-inflammatory effects of GRS appeared to be more evident under AQP4-expressing conditions. This interpretation is supported by the observation that TGN-020, a reference AQP4 functional inhibitor, attenuated LPS- and TNF-α-induced KC mRNA expression and ERK phosphorylation in a manner similar to GRS (Figures 1 and 2E). In addition, GRS did not clearly suppress inflammatory responses in EV-transfected NIH-3T3 cells, which showed little endogenous AQP4 expression, whereas its suppressive effects were observed under AQP4-expressing conditions (Figure 2). In contrast, DEX suppressed inflammatory responses in both EV- and AQP4-transfected cells. These results suggest that GRS-responsive inflammatory signaling differs from that affected by a general anti-inflammatory agent such as DEX

AQP4 expression was also associated with greater ERK activation in response to inflammatory stimulation. In the present study, U0126, a specific inhibitor of ERK signaling, attenuated the increase in KC mRNA expression observed in AQP4-expressing cells, with less evident effects in EV-transfected cells (Figure 3B). In addition, Cinnamomi cortex, cinnamaldehyde, cinnamic acid, and GRS attenuated ERK phosphorylation under AQP4-expressing conditions, which was consistent with the changes in KC mRNA expression (Figures 3, 4, 5).

In the present study, LPS and TNF-α were used for different experimental purposes. Intracerebroventricular LPS administration was used to induce an acute and broad neuroinflammatory response involving multiple cell types and inflammatory mediators, thereby allowing evaluation of orally administered GRS in an integrated brain environment. In contrast, TNF-α was used as a defined inflammatory stimulus in the screening experiments to compare the effects of individual crude drugs and chemical constituents under simplified conditions. Although LPS and TNF-α initiate signaling through different receptors, TLR4 and TNF receptors, respectively, their downstream inflammatory pathways partially overlap, including MAPK/ERK and NF-κB signaling. Moreover, AQP4 expression enhanced chemokine mRNA expression following both LPS and TNF-α stimulation, and GRS suppressed these responses under both conditions (Supplementary Data sheet 1). Thus, the TNF-α-stimulated system was considered suitable for identifying crude drugs and constituents contributing to the effects of GRS. Nevertheless, these findings do not demonstrate that the identified crude drugs or constituents act directly on the LPS–TLR4 pathway or fully explain the effects of GRS in the *in vivo* LPS model. Rather, the consistent effects observed with both stimuli suggest that GRS and its constituents may modulate downstream inflammatory processes shared, at least in part, by LPS and TNF-α.

Previous pharmacokinetic studies have reported that cinnamic acid and related compounds derived from Cinnamomi cortex can penetrate into the brain (Hida et al., 2021). Therefore, some constituents of Goreisan may reach the central nervous system and exert pharmacological effects within the brain. However, because GRS is a complex herbal formulation containing multiple bioactive constituents.

In addition to its role in inflammatory amplification, AQP4 is a core component of the glymphatic system, mediating the clearance of inflammatory cytokines, metabolic waste products, and amyloid-β (Ding et al., 2023; Iliff et al., 2012). Although excessive or mislocalized AQP4 expression may contribute to pathological inflammation, physiological AQP4 activity is essential for maintaining efficient glymphatic function (Iliff et al., 2014; Kress et al., 2014). Thus, any therapeutic strategy involving AQP4 should consider its dual role in supporting cerebrospinal–interstitial fluid exchange and amplifying neuroinflammation under pathological conditions.

A limitation of the present study is that cell viability and cytotoxicity were not quantitatively evaluated in primary astrocytes or NIH-3T3 cells; therefore, a contribution of mild cytotoxicity to the observed reductions in inflammatory gene expression cannot be excluded. In addition, because cytokines and chemokines were primarily evaluated at the mRNA level, the present findings do not establish corresponding changes in protein production or release. The data also do not identify AQP4 as the direct or primary molecular target of GRS, and AQP4-independent mechanisms may contribute to its effects. Further studies assessing cell viability, inflammatory mediator production at the protein level, and the direct involvement of AQP4 are required.

Taken together, these findings suggest that GRS attenuates inflammatory gene expression and glial activation, potentially through mechanisms involving AQP4-related ERK signaling and suppression of inflammation-associated AQP4 upregulation. Although further studies are required to clarify the direct contribution of AQP4 functional inhibition and to confirm cytokine and chemokine release at the protein level, modulation of AQP4-related inflammatory responses may partly explain the pharmacological effects of GRS. These findings may provide a basis for future studies investigating the potential relevance of GRS in neuroinflammatory conditions in which AQP4 and glial activation are implicated, such as neuromyelitis optica spectrum disorder, multiple sclerosis, Alzheimer’s disease, and headache.

## Data Availability

The raw data supporting the conclusions of this article will be made available by the authors, without undue reservation.
