# Supplementary material for: Goreisan-mediated suppression of chemokine and cytokine production in astrocytes under AQP4-expressing conditions
Source: Front Neurosci. 2026 Aug 31;20:1860290. doi: 10.3389/fnins.2026.1860290 (PMC13572713; doi:10.3389/fnins.2026.1860290)
Supplement: Supplementary file 1 [file Data_Sheet_1.docx]

Supplementary Material

## Supplementary Figures

**Supplementary Figure 1.** **GRS suppressed LPS-induced AQP4 expression in primary cultured astrocytes**
Primary cultured astrocytes were stimulated with LPS (100 ng/ml) in the presence or absence of Goreisan (GRS; 0.01–0.1 mg/ml). The mRNA expression of AQP4 was measured by RT-qPCR.
Data are presented as mean ± SEM (n = 3). Statistical analysis was performed using a one-way analysis of variance (ANOVA), followed by Tukey’s multiple comparison test.
^#^P < 0.05 vs. control; *P < 0.05 vs. LPS-treated group.

**Supplementary Figure 2. Dose- and time-dependent effects of GRS on TNF-α-induced chemokine expression in NIH-3T3 cells.**
NIH-3T3 cells were transfected with AQP4 or empty vector (EV) and stimulated with TNF-α (40 ng/ml). (A) Cells were treated with increasing concentrations of Goreisan (GRS; 0.01–0.1 mg/ml). (B) Cells were treated with GRS (0.1 mg/ml) for the indicated time periods in the presence or absence of TNF-α. KC mRNA expression was measured by RT-qPCR.
Data are presented as mean ± SEM (n = 3). Statistical analysis was performed using two-way ANOVA followed by Tukey’s multiple comparison test.
^#^P < 0.05 vs. EV-control; *P < 0.05 vs. TNF-α-treated group.
